# Physical activity based interventions for reducing body mass index in children aged 6–12 years: a systematic review

**DOI:** 10.3389/fped.2025.1449436

**Published:** 2025-07-18

**Authors:** Nicolás Muñoz-Urtubia, Alejandro Vega-Muñoz, Guido Salazar-Sepúlveda, Miguel Ángel García-Gordillo, José Carmelo-Adsuar

**Affiliations:** ^1^ Facultad de Filosofía y Humanidades, Universidad Austral de Chile, Valdivia, Chile; ^2^ Escuela Internacional de Graduados, Universidad de Extremadura, Cáceres, Spain; ^3^ Centro de Investigación en Educación de Calidad Para la Equidad, Universidad Central de Chile, Santiago, Chile; ^4^ Facultad de Ciencias Empresariales, Universidad Arturo Prat, Iquique, Chile; ^5^ Facultad de Ingeniería, Universidad Católica de la Santísima Concepción, Concepción, Chile; ^6^ Facultad de Ingeniería y Negocios, Universidad de las Américas, Concepción, Chile; ^7^ Facultad de Ciencias Jurídicas y Empresariales, Universidad de La Frontera, Temuco, Chile; ^8^ Facultad de Ciencias del Deporte, Universidad de Extremadura, Cáceres, Spain

**Keywords:** body mass index, obesity, prevalence, early intervention, physical activity

## Abstract

**Introduction:**

This systematic review aimed to examine the impact of physical activity-based interventions on body mass index (BMI) reduction in children aged 6–12 years.

**Methods:**

A comprehensive search was conducted in the PubMed database following PRISMA guidelines and using the PICOS framework. A total of 13,927 records were retrieved, of which seven studies met the inclusion criteria. Methodological quality was assessed using the Mixed Methods Appraisal Tool (MMAT).

**Results:**

Included studies comprised both preventive and treatment-oriented interventions that aimed to reduce BMI through physical activity. Interventions that integrated family and school components, and were grounded in behavioral theories such as Self-Determination Theory and Social Cognitive Theory, showed greater effectiveness in reducing BMI and improving body composition.

**Discussion:**

Multilevel strategies that enhance autonomy, competence, and social support within biopsychosocial frameworks appeared to improve motivation and adherence. Although BMI z-score reductions were modest, they reached clinically meaningful thresholds. These findings support the implementation of context-sensitive, comprehensive strategies involving families, schools, and communities to promote healthy behaviors and sustainable outcomes in pediatric populations.

**Systematic Review Registration:**

https://www.crd.york.ac.uk/prospero/, PROSPERO CRD42024547428.

## 1 Introduction

Pediatric obesity is a multifactorial disease influenced by biological, psychological, social and environmental factors (1, 2). Factors such as genetic predisposition, poor diet, sedentary lifestyle, low physical activity, mental health problems, socioeconomic context and family dynamics influence both its development and persistence (3, 4). Understanding pediatric obesity requires a biopsychosocial perspective that considers the complexity of interactions among all its causes (5, 6). Physical activity-based interventions, although the focus of this review, show greater efficacy when framed within comprehensive lifestyle and environmental changes (7).

In this context, obesity is currently regarded as a global health problem due to its relentless growth and high prevalence. Consequently, the rates of childhood overweight and obesity have increased significantly worldwide (8). The World Health Organization (WHO) classifies it as a global pandemic (9, 10), making it an urgent public health issue (10, 11). This situation can trigger various pathologies associated with sedentary lifestyles, with lifelong repercussions (12, 13).

Most health issues associated with a sedentary lifestyle are linked to obesity (14). A particularly noteworthy factor contributing to the rising prevalence of overweight and obesity in children and adolescents is the low rate of physical activity observed in this age group. This is largely due to the extended periods spent sitting during school classes (15, 16) and in front of digital screens (17–19). It is estimated that approximately 80% of children and adolescents worldwide do not meet the minimum recommendations for moderate to vigorous physical activity (MVPA) as set out by the WHO (10, 17, 20).

Overweight and obesity, which are associated with high levels of adiposity, have serious health consequences. These include cardiovascular problems, respiratory problems, and metabolic diseases (21, 22). In addition, they can also negatively affect psychosocial and cognitive health (23, 24). Overweight or obese children are more likely to become adults with the same health problems (10). As children grow older, their physical activity levels tend to decrease, which generates a vicious circle between low physical activity and increased adiposity (17, 25, 26).

The benefits of physical activity in childhood and adolescence are well documented. These benefits include improved body composition (27, 28), increased muscle strength and power (29, 30), increased bone mineral density (30, 31), and development of basic motor skills (32, 33). In addition, physical activity reduces the risk of musculoskeletal injuries (33, 34), improves cardiovascular health (35), and increases insulin sensitivity (27, 36).

Likewise, there are academic and social benefits associated with higher levels of cardiorespiratory fitness and cognitive development in children and adolescents (37, 38). It is therefore of paramount importance to maintain and promote physical activity in this population, as physical fitness is an important indicator of overall health (39–42).

Finally, because the higher body mass index in children and adolescents is associated with higher levels of physical inactivity (19, 24, 43), the aim of this review is to analyse the impact of different types of interventions on reducing BMI in the pediatric population and to identify strategies to improve body composition in children. This will allow the design of better prevention and health promotion approaches in this age group.

Given the above, childhood obesity is a public health challenge that requires effective interventions, with physical activity being a key strategy. From a biopsychosocial perspective, which integrates biological, psychological and social factors, it becomes fundamental to consider not only body mass index (BMI), but also body composition as indicators of change. Furthermore, the effectiveness of these interventions depends on contextual and motivational elements such as family and school participation, the duration and intensity of the programs, and the use of technologies. In this framework, it is relevant to identify which characteristics of physical interventions are associated with better outcomes in the childhood population. Which characteristics of physical activity-based interventions, framed within a biopsychosocial approach, are associated with greater reductions in BMI and improvements in body composition in the childhood population?

## 2 Methods

The systematic review was registered in the PROSPERO database (CRD42024547428). Available at https://www.crd.york.ac.uk/prospero/. PRISMA guidelines were used for this review (44) and PICOS strategies were used to establish eligibility criteria for the articles (45). The review was performed in PubMed, a free specialized database in biomedicine and health, ensuring that the selected articles were directly related to the topic of interest. In addition, the metadata of the PubMed database allows the incorporation of specific search filters such as: Article type, Species, and Age, in this case Randomized Controlled Trial, Human and Child: 6–12 years.

The use of PubMed in biomedical and health systematic reviews is essential due to its high subject specificity, open access and comprehensive coverage of peer-reviewed literature in medical sciences. Studies such as those by Falagas et al. (46) and AlRyalat et al. (47) highlight that PubMed offers more accurate and relevant searches compared to broader databases such as Scopus or Web of Science, mainly thanks to its indexing system with MeSH terms that optimizes the retrieval of specific information, as well as to the specific metadata that is incorporated into the reported studies, such as: clinical typology, species (human and animal), and age range. In addition, recent research such as Kokol (48) evidence important discrepancies in coverage and funding information between Scopus and WoS, underscoring the need to complement these databases with PubMed to obtain a more complete and reliable view. Therefore, incorporating PubMed guarantees methodological rigor and thematic exhaustiveness, essential elements for the quality of systematic reviews in the field of health.

### 2.1 Search strategies and data sources

The search was performed in the PubMed electronic database using the search vector with the terms: [body mass index (MeSH Terms)] AND [activity, physical (MeSH Terms)]. The search was limited to articles published since 2020 and was conducted on May 16, 2024. Only articles written in English, which accounts for over 95% of indexed publications, and that were randomized clinical trials (RCTs) were included and gray literature was not considered.

### 2.2 Eligibility criteria

The selection of articles was based on the PICOS criteria, as shown in Table 1. Only randomized, controlled studies involving pediatric population were included, focusing on the reduction and measurement of participants' BMI. Studies were included regardless of whether the intervention included family, school, or both components. Although the inclusion of these contexts was not a requirement for selection, their presence or absence was subsequently documented and analyzed as a potential factor influencing intervention outcomes. Eligible studies focused on the promotion of healthy lifestyles in children through structured physical activity interventions. Studies involving pharmacological, surgical, or exclusively nutritional approaches were excluded. Studies could target either the prevention or treatment of overweight and obesity, as long as the primary outcome was a change in BMI and the intervention included a physical activity component. This review focused on children aged 6–12 years, as this developmental period is characterized by greater stability in behavioral patterns and body composition compared to early childhood or adolescence, making it an ideal window for implementing and evaluating structured physical activity interventions (49, 50).

**Table 1:** Eligibility criteria using PICOS (participants, interventions, comparators, outcomes, and study design).

PICOS	Description
Participants	Children participating in interventions designed for BMI modification.
Interventions	Exercise, sports, school physical education, and activities involving family and school to promote healthy lifestyles.
Comparators	Groups with no intervention, different interventions, or standard physical activity programs.
Outcomes	BMI reduction, improved body composition, general health, and adherence to physical activity.
Study Design	Randomized clinical trials (RCT) that were included according to the quality criteria of the MMAT scale (51).

### 2.3 Study selection, data extraction and quality assessment

The selection process was carried out in two stages. In the first stage, two independent reviewers evaluated the titles and abstracts of the studies obtained through the search strategy to determine their relevance. Studies that did not meet the inclusion criteria were excluded. Then, in the second stage, the pre-selected studies were subjected to a full text review by the same reviewers to confirm their eligibility. Discrepancies between reviewers were resolved by the intervention of a third reviewer.

Subsequently, details of the interventions and the control group were extracted. This data extraction was performed independently by two reviewers to ensure accuracy. The results obtained were compared and any discrepancies were resolved by discussion or consultation with a third reviewer.

The MMAT (Mixed Methods Appraisal Tool) scale for RCT was applied during the selection and evaluation of studies by three independent reviewers to guarantee quality and methodological rigor, the third being consulted in the event of any discrepancy. The MMAT scale is a checklist based on the synthesis of qualitative and quantitative evidence that includes criteria for the evaluation of mixed studies. The study category is defined, and 7 items are applied according to a score from 0 to 1, obtaining a final percentage measure. Studies are considered to be of high quality >75%, medium quality between 50% and 74% and low quality <49% (51). Of the studies included in this review, only one was of medium quality according to the MMAT scale.

## 3 Results

**Figure 1:**
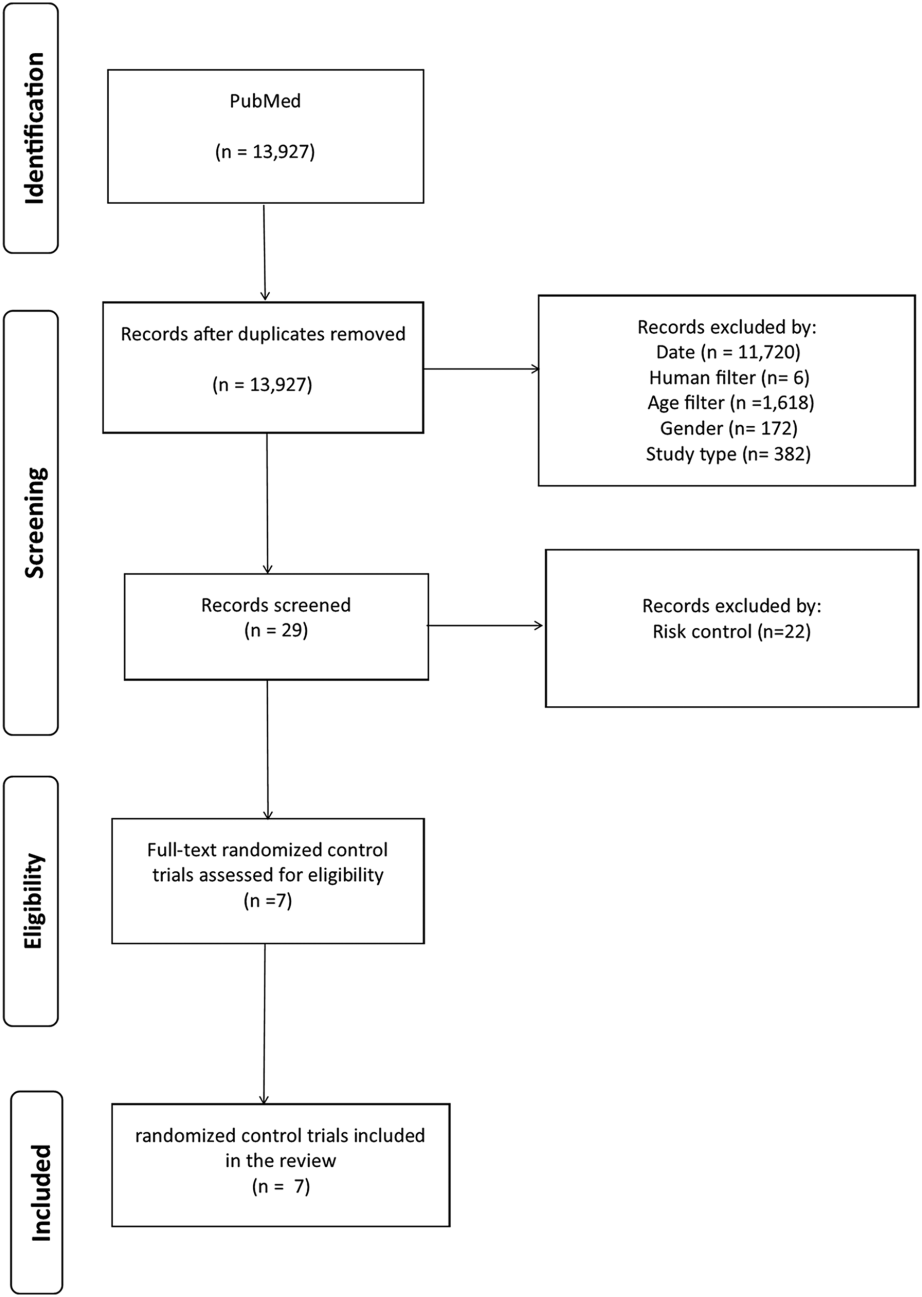
Preferred reporting elements analysis flow for systematic reviews and meta-analyses (PRISMA).

The initial PubMed search vector extracted a total of 13,927 records. By applying inclusion filters, such as publication date since 2020, human species, age 6–12 years, gender, study type, and risk control, records are lost at each screening stage. In total, 11,720 records are lost by date, 6 by human species, 1,618 by age, 172 by gender (male, female), 382 by study type, and 22 by risk control. At the end of this process, only articles that meet all inclusion criteria are selected, resulting in a final number of 7 articles for the systematic review (see Figure 1).

Using the PRISMA guidelines, 7 articles were selected (44). Table 2 shows the details, which are authors, journal, year of publication, citation, indexing, sample and study design. In addition, Table 3 details the eligibility criteria of the articles using the MMAT scale.

**Table 2:** Characteristics of selected studies.

Authors	Journal	Publication year	Sample	Intervention	Study Duration	zBMI (%BF Reduction)	Main Outcome	Pubmed (Y/N)	Times Cited	Category of Study Design
Liu et al. (52)	JAMA Pediatr	8–10	*n* = 1,362* (IG = 686 CG = 676) *30 excluded due to follow-up loss	Multicomponent school PA	12 months	ZBMI: −0.17	Significant ZBMI and obesity reduction	Y	27	Randomized control trials
Diao et al. (11)	Health Qual Life Outcomes	9–11	*n* = 948 (IG = 518 CG = 430)	PA + education + follow-up	12 months	ZBMI: −0.24	Improved QoL and ZBMI	Y	10	Randomized control trials
Comeras-Chueca et al. (10)	J Environ Res Public Health	11–13	*n* = 29 (AVG = 21 CG = 8)	HIIT + strength	5 weeks	ZBMI: −0.35; ↓ %BF	Significant body composition improvement	Y	14	Randomized control trials
Fulkerson et al. (53)	J Behav Nutr Phys Act	7–10	*n* = 102 (IG = 56 CG = 46)	Family-based intervention	7 months	NS ZBMI; ↓ %BF (boys)	Effect in %BF among boys only	Y	7	Randomized control trials
Raynor et al. (54)	Child Obes	4–10	*n* = 73 (IG = 35 CG = 38)	PP + (with goals) vs. PP−	6–9 months	PP+: −0.20; PP−: −0.05	Only PP + achieved clinical ZBMI reduction	Y	1	Randomized control trials
Long et al. (55)	BMC med	6–12	*n* = 1,304* (PA = 327 PA + MMNS = 286 MMNS = 305 CG = 309) *77 excluded due to missing data	School PA + MMNS	9 months	↓ FM and TrFM (girls)	Effects by sex and growth	Y	5	Randomized control trials
Varagiannis, et al. (56)	Nutrients	8–12	*n* = 115 g1 = 36 g2 = 30 g3 = 25* * 24 excluded due to droput	3 family interventions (group, individual, web)	6 months	ZBMI ↓ in all; %BF: −2.1% (web), −1.9% (individual)	Individualized intervention most effective	Y	9	Randomized control trials

**Table 3:** Eligibility criteria using the mixed methods assessment tool (MMAT).

Authors	Journal	Publication year	Category of study design	S1	S2	2.1	2.2	2.3	2.4	2.5	Quality
Liu et al. (52)	JAMA Pediatr	2021	Quantitative RCT	1.0	1.0	1.0	1.0	1.0	1.0	1.0	100%
Diao et al. (11)	Health Qual Life Outcomes	2020	Quantitative RCT	1.0	1.0	1.0	1.0	1.0	1.0	1.0	100%
Comeras-Chueca et al. (10)	J Environ Res Public Health	2022	Quantitative RCT	1.0	1.0	1.0	1.0	1.0	0.0	1.0	86%
Fulkerson et al. (53)	J Behav Nutr Phys Act	2022	Quantitative RCT	1.0	1.0	0.0	1.0	1.0	1.0	1.0	86%
Raynor et al. (54)	Child Obes	2022	Quantitative RCT	1.0	1.0	1.0	1.0	0.0	1.0	1.0	86%
Long et al. (55)	BMC med	2022	Quantitative RCT	1.0	1.0	1.0	1.0	0.0	1.0	1.0	86%
Varagiannis, et al. (56)	Nutrients	2021	Quantitative RCT	1.0	1.0	1.0	1.0	0.0	0.0	1.0	71%

The heterogeneity of the results reported in Table 2 prevents progressing towards a meta-analytic analysis. Thus, the studies included in this review were classified according to their main themes into 4 categories as shown in Table 4.

**Table 4:** Classification according to main themes in 4 categories.

Authors	HP	MMI	IMC-A	TEC	Total
Liu et al. (52)	X	X	X	-	3
Diao et al. (11)	X	X	X	-	3
Comeras-Chueca et al. (10)	X	-	X	X	3
Fulkerson et al. (53)	X	X	X		3
Raynor et al. (54)	X	X	X	X	4
Long et al. (55)	X	X	X	-	3
Varagiannis, et al. (56)	X	X	X	X	4
Total	7	6	7	3	

From the articles analyzed, 4 total categories concerning effective interventions related to the decrease of BMI in pediatric population emerged, which are: Health promotion and healthy behavior modification (HP), Integral and multidimensional interventions (MMI), Use of BMI as an indicator of adiposity in pediatric population (BMI-A), Incorporation of technologies in intervention and follow-up (TEC). The results are detailed below.

### 3.1 Health promotion and healthy behavior modification

Five of the categorized articles focused on interventions involving the family and/or caregivers, the school, or both (11, 52–55). Health promotion and healthy behavior modification focus mainly on educational strategies and programs that promote and shape healthy lifestyle habits in the pediatric population. These include education on balanced diet, promotion of regular physical activity, reduction of screen time and setting nutritional goals for caregivers and families as well as for children and adolescents participating in the programs. Early adoption of lifestyle changes is crucial, since when they are adopted at an early age, they tend to be more stable throughout the different stages of growth.

### 3.2 Integral and multidimensional interventions

Integral and multimodal interventions include the coordinated participation of different stakeholders, such as family, caregivers, teachers, and the school community at large. These interventions are more effective in improving adherence to BMI reduction interventions in the pediatric population compared to those that do not involve multiple stakeholders. Comprehensive, multidimensional interventions create a holistic supportive environment that facilitates the adoption and maintenance of healthy behaviors through a consistent and cohesive support network. This approach recognizes the importance of a structured and supportive environment that includes not only the immediate family, but also other important stakeholders in the child's life, such as teachers and health professionals, thus ensuring more effective and sustained implementation of lifestyle changes.

### 3.3 Use of BMI as an indicator of adiposity in the pediatric population

In all the articles reviewed, BMI was used as the gold standard to measure the general adiposity of the subjects. Although one study supplemented BMI data with bioimpedance (56), BMI is confirmed as a reliable tool to assess the risk of being overweight and obesity in childhood. It stands out as a valid option for measuring body fat in the pediatric population, as the poor development of muscle mass at this stage suggests that increases in body mass are mainly due to increased fat mass (57).

### 3.4 Incorporation of technologies in intervention and follow-up

The use of digital technologies and technological tools facilitates the implementation of interventions as well as monitoring and follow-up during these interventions aimed at reducing BMI in children and adolescents. Within this category were included the use of active video games and accelerometer (10), reports through digital forms (54), use of mobile applications (52) and web pages (56). The inclusion of technology can increase participation, since the tools used allow for more accurate measurement of progress, in addition to generating greater attraction for participants to increase daily physical activity.

## 4 Discussion

### 4.1 Framing interventions within a biopsychosocial perspective

The findings of this review underscore that physical activity-based interventions are more effective when framed within a biopsychosocial approach, which incorporates family dynamics, school settings, individual behaviors, and broader social contexts, with families acting not just as contextual modulators, but as active agents whose engagement is central to intervention success.

This integrative perspective is exemplified by Liu et al. (52), whose intervention included both school and family environments through app-based follow-up, emphasizing the influence of parental involvement and environmental support in facilitating behavior change.

This aligns with Engel's biopsychosocial model (5), which posits that complex health outcomes like pediatric obesity result from the interplay between biological, psychological, and social factors. Applying this framework enhances the interpretative power of the current findings.

The review synthesizes evidence supporting the efficacy of physical activity-based interventions in reducing BMI in children aged 6–12 years. While it included both preventive and treatment-focused strategies, the distinction between these two approaches was not a central analytical criterion. For instance, Fulkerson et al. (53) conducted a preventive intervention targeting a general population, whereas Varagiannis et al. (56) focused on treating children with overweight or obesity.

While this review included both preventive and treatment-oriented interventions, few studies clearly specified their primary intent or described baseline characteristics in detail. This lack of differentiation limited the ability to assess outcome variations across subgroups.

Notably, Raynor et al. (54) did report baseline BMI classifications and found that clinically meaningful reductions in BMI *z*-scores were achieved only in the group involving caregiver goal setting. This suggests that personalized, family-inclusive approaches may be particularly effective in treatment settings.

These distinctions are relevant because children with overweight or obesity at baseline may respond differently than their normal-weight peers, particularly when the intensity and behavioral support of the intervention vary.

### 4.2 Motivational mechanisms and theoretical foundations

According to Self-Determination Theory (58), intrinsic motivation is strengthened when individuals experience autonomy, competence, and social connectedness. Interventions that support these factors are more likely to foster lasting behavioral change, particularly when tailored to the developmental stage of children and embedded in supportive environments.

This approach is exemplified by Liu et al. (52), whose intervention combined family engagement with school-based activities to enhance environmental and social support.

Supporting this framework, a recent study in Chilean schoolchildren (59) found a significant inverse relationship between BMI and motivation for physical activity. These findings suggest that interventions focusing on motivational components, particularly within educational settings, may yield more effective outcomes. They also underscore the need to go beyond informational strategies and incorporate behavioral reinforcement mechanisms.

Additionally, Bandura's Social Cognitive Theory (60) emphasizes the importance of perceived self-efficacy, observational learning, and social reinforcement in adopting and maintaining health-related behaviors.

Applying this lens, interventions that incorporate role models, parental reinforcement, and peer support may further enhance behavioral adherence, a crucial determinant of long-term outcomes.

While theoretical models such as Self-Determination Theory and Social Cognitive Theory provide a strong foundation for understanding motivation, their application is most effective when combined with active family involvement that reinforces autonomy, competence, and behavioral modeling at home.

### 4.3 Intervention intensity, duration, and adherence

The included studies exhibited considerable variability in terms of intensity and duration, ranging from short-term interventions to year-long strategies. For example, Comeras-Chueca et al. (10) implemented a 5-week high-intensity program focused on HIIT and strength training, while Liu et al. (52) conducted a 12-month intervention integrating school and family components.

Despite these differences, most studies failed to report the total number of contact hours. None appeared to meet the 26-hour minimum recommended by the U.S. Preventive Services Task Force (USPSTF) for effective pediatric obesity treatment (61). This omission limits the clinical applicability of the findings, as it weakens alignment with established guidelines. The discrepancy between research protocols and clinical standards may also help explain the modest effect sizes observed, reinforcing the need for more intensive, sustained interventions.

Intervention effectiveness was influenced by several key factors, including participant adherence, program continuity, measurement reliability, and the involvement of multiple stakeholders in children's health.

The most successful interventions in reducing BMI and preventing obesity were those that engaged both schools and caregivers. For instance, Jacob et al. (62) emphasized the critical role of family and teacher involvement in improving pediatric BMI. Furthermore, Raynor et al. (54) found that when caregivers were encouraged to set and achieve their own nutritional goals, children's body composition improved even more. These results also align with the recommendations of the WHO Commission on Ending Childhood Obesity, which advocates for school-based and family-centered strategies as core elements of national public health plans aimed at reducing childhood obesity (63).

### 4.4 Effectiveness and clinical significance of BMI reductions

Across the studies included in this review, reductions in BMI *z*-scores ranged from −0.17 to −0.35. These values align with prior evidence indicating that even small improvements (≥ −0.1 ZBMI) are associated with clinically meaningful reductions in cardiometabolic risk in children (64).

In addition to BMI changes, some studies reported improvements in body composition. For example, Raynor et al. (54) and Varagiannis et al. (56) documented decreases in total body fat percentage ranging from 1.9% to 2.1%, reinforcing the clinical significance of these interventions, even in short-term formats.

Taken together, the findings suggest that even modest BMI reductions achieved through physical activity-based interventions supported by families and schools are clinically relevant and contribute meaningfully to pediatric obesity prevention within a biopsychosocial framework.

These modest yet clinically meaningful reductions, particularly when supported by families and schools, underscore the value of biopsychosocial strategies in sustainable pediatric obesity prevention.

The role of schools in addressing childhood obesity is especially relevant given current behavioral patterns. Children and adolescents today spend prolonged periods sitting, particularly during school hours, and increasingly engage in screen-based leisure activities (8, 9, 65).

These behavioral trends contribute to low levels of physical activity worldwide. According to global surveillance data, approximately 80% of children and adolescents fail to meet the World Health Organization's recommendation of at least 60 min of moderate-to-vigorous physical activity per day (17, 20). This lack of adherence to basic activity guidelines underscores the need for school-based programs that can interrupt sedentary routines and promote active behaviors throughout the day.

On the other hand, improving BMI outcomes in children requires promoting healthy behaviors beyond physical activity alone. Key strategies include increasing the consumption of fruits and vegetables (66), reducing intake of sugar and ultra-processed foods (67), limiting sedentary and screen time (62, 68), and promoting daily movement (69).

Reviews such as Tremblay et al. (65) support the effectiveness of reducing screen time to under 2 h per day in combination with increasing daily physical activity. However, as highlighted by Spiga et al. (70), dietary interventions alone are generally insufficient to achieve long-term BMI improvements. This reinforces the need for integrated strategies that combine nutritional education with physical activity promotion.

Different approaches have proven useful in increasing MVPA in children, including traditional methods such as aerobic and strength training (71, 72), high-intensity interval training (HIIT) (73, 74), and even active video games. Although not a substitute for structured exercise, these games can complement programs by increasing daily energy expenditure, as suggested by Comeras-Chueca et al. (10). Jurado-Castro et al. (75) further emphasize that increasing daily MVPA should be a central strategy for reducing general adiposity in the pediatric population.

### 4.5 Limitations of BMI and the need for complementary measures

In addition, despite its limitations, BMI remains a widely used and practical tool for assessing adiposity and obesity risk in pediatric populations. Its consistent use across studies enables comparisons between different groups and settings (70, 76).

Notably, BMI has also demonstrated utility in children with specific conditions such as Down syndrome and autism spectrum disorder. Although these populations may not show significant BMI reductions following interventions, studies indicate that BMI can still serve as a valid indicator for monitoring body composition changes and health risks (77, 78).

These findings support the continued use of BMI as a baseline measure in pediatric health research and practice. However, they also highlight the importance of complementing BMI with other indicators, such as fat percentage, lean mass, or bioimpedance análisis, to better capture the nuanced physiological effects of interventions.

### 4.6 Multilevel approaches and the role of stakeholders

Our findings confirm that multimodal interventions, those involving families, caregivers, schools, and health professionals, are the most effective for reducing BMI in children. These approaches benefit from the interaction of multiple stakeholders who influence children's daily behaviors and environments.

The integration of technological tools into such interventions has also shown promise. Wearables, mobile applications, and digital platforms can enhance adherence, enable individualized feedback, and improve monitoring capacity. When combined with in-person support and structured routines, these tools may help sustain healthy behaviors over time.

BMI continues to be a viable and widely used indicator for assessing adiposity in pediatric populations; however, its interpretation should prioritize changes in adipose tissue rather than BMI values alone (79). To enhance accuracy, it is recommended to complement BMI with additional assessments such as bioimpedance analysis (13). Furthermore, effective interventions should integrate educational strategies that promote healthy behavior adoption, involving not only schools, parents, and students, but also health professionals. Given that overweight and obesity constitute pressing public health concerns (11), incorporating healthcare system actors is essential for achieving broader and more sustained outcomes (53).

Childhood obesity remains a global public health concern, with significant physical, psychological, and social consequences. School-based interventions have shown particular effectiveness in addressing this issue, especially when multiple strategies are combined.

### 4.7 Evidence from diverse sociocultural contexts

In China, Liu et al. (52) implemented a multifaceted intervention that included educational activities, modifications to the school environment, and family involvement. This approach led to substantial improvements in anthropometric indicators and health-related behaviors. Similarly, Diao et al. (11) reported that multi-component interventions not only reduced body weight but also enhanced health-related quality of life among children and adolescents.

Evidence from other settings supports these findings. In the United States, the NU-HOME study conducted in rural communities by Fulkerson et al. (53) demonstrated that a family-centered intervention aimed at improving the home food environment and promoting physical activity produced positive outcomes in weight management. Likewise, Raynor et al. (54) showed that implementing the Prevention Plus program in underserved primary care settings was both feasible and well received, emphasizing the importance of tailoring interventions to sociocultural and economic contexts.

In the Global South, Long et al. (55) evaluated the KaziAfya program in South Africa and found that comprehensive school-based strategies, including regular physical activity and health education, effectively improved children's body composition. This approach underscores the school as a key setting for instilling healthy behaviors from an early age, particularly when families, educators, and health professionals are actively involved.

School-based interventions appear most effective when they combine educational content, structural changes, and active community participation. To ensure sustainability and long-term impact, such interventions must also account for cultural, geographic, and socioeconomic diversity.

Taken together, the findings of this review highlight that physical activity-based interventions, particularly those integrating family and school components, and supported by motivational and behavioral strategies, are most effective in promoting BMI reduction in children. To ensure long-term sustainability, such interventions must be embedded within biopsychosocial frameworks that account for cultural, geographic, and socioeconomic diversity. Integrative models that adapt to specific community needs enhance engagement, relevance, and effectiveness, making them key to scalable and equitable public health strategies.

### 4.8 Implications for future research and public health

A key limitation of this review is the exclusive use of PubMed as the search database, which may have restricted the scope of included studies, as relevant research indexed in other databases such as Scopus or Web of Science might have been omitted. Although the selection process was rigorous, only seven studies were ultimately included from over 13,000 records, which may limit the generalizability of the findings. Future systematic reviews should incorporate additional databases to enhance comprehensiveness and minimize potential selection bias.

In addition, future research should expand beyond BMI as a sole indicator and incorporate additional measures, such as fat percentage, muscle mass, or waist circumference, to more accurately reflect changes in body composition. Longitudinal studies are also needed to assess the sustained impact of interventions over time. Moreover, incorporating digital tools could enhance adherence and support the adoption of healthy behaviors, particularly in children and adolescents, where early prevention and health promotion have long-term benefits.

## 5 Conclusion

This systematic review confirms that physical activity-based interventions can reduce BMI and improve body composition in children aged 6–12 years, particularly when both families and schools are actively involved. Multilevel strategies that engage caregivers strengthen adherence and motivation through consistent social and environmental support.

While BMI remains a practical tool in pediatric health, its limitations, especially during growth and puberty, highlight the need for complementary indicators such as fat percentage, lean mass, and waist circumference to more accurately reflect physiological changes.

Interventions grounded in Self-Determination Theory and Social Cognitive Theory appear more effective in promoting autonomy, competence, and social connectedness. However, sustained outcomes are most likely when these frameworks are embedded within a broader biopsychosocial perspective that includes families, schools, and community environments.

Although the observed reductions in BMI *z*-scores were modest, they fall within clinically meaningful thresholds and may contribute to improved cardiometabolic health. Still, the short duration and limited intensity of most interventions indicate the need for more robust, long-term strategies aligned with clinical guidelines.

In conclusion, public health strategies should prioritize comprehensive, context-sensitive physical activity programs that engage families, schools, and communities, not only to reduce excess weight, but also to promote physical literacy, healthy behaviors, and psychosocial well-being.

## Data Availability

The original contributions presented in the study are included in the article/Supplementary Material, further inquiries can be directed to the corresponding author.
