# Supplementary material for: Physical activity based interventions for reducing body mass index in children aged 6–12 years: a systematic review
Source: Front Pediatr. 2025 Jul 18;13:1449436. doi: 10.3389/fped.2025.1449436 (PMC12313624; doi:10.3389/fped.2025.1449436)
Supplement: Supplementary file 1 [file Table1.docx]

**Physical Activity Based Interventions for Reducing Body Mass Index in Children Aged 6 to 12 years: A Systematic Review**

Supplementary Material: 29 Records Screened.

1. Jerome GJ, Fink T, Brady T, Young DR, Dickerson FB, Goldsholl S, Findling RL, Stepanova EA, Scheimann A, Dalcin AT, Terry A, Gennusa J, Cook C, Daumit GL, Wang NY. Physical Activity Levels and Screen Time among Youth with Overweight/Obesity Using Mental Health Services. Int J Environ Res Public Health. 2022 Feb 17;19(4):2261. doi: 10.3390/ijerph19042261. PMID: 35206449; PMCID: PMC8871648.
2. Liu Z, Gao P, Gao AY, Lin Y, Feng XX, Zhang F, Xu LQ, Niu WY, Fang H, Zhou S, Li WH, Yuan JH, Xu CX, Wu N, Li HJ, Wen LM, Patton GC, Wang HJ, Wu YF. Effectiveness of a Multifaceted Intervention for Prevention of Obesity in Primary School Children in China: A Cluster Randomized Clinical Trial. JAMA Pediatr. 2022 Jan 1;176(1):e214375. doi: 10.1001/jamapediatrics.2021.4375. Epub 2022 Jan 4. PMID: 34747972; PMCID: PMC8576631.
3. Comeras-Chueca C, Villalba-Heredia L, Perez-Lasierra JL, Marín-Puyalto J, Lozano-Berges G, Matute-Llorente Á, Vicente-Rodríguez G, Gonzalez-Aguero A, Casajús JA. Active Video Games Improve Muscular Fitness and Motor Skills in Children with Overweight or Obesity. Int J Environ Res Public Health. 2022 Feb 24;19(5):2642. doi: 10.3390/ijerph19052642. PMID: 35270330; PMCID: PMC8910272.
4. Bjøntegaard KA, Stafne SN, Mørkved S, Salvesen KÅ, Evensen KAI. Body mass index and physical activity in seven-year-old children whose mothers exercised during pregnancy: follow-up of a multicentre randomised controlled trial. BMC Pediatr. 2021 Nov 8;21(1):496. doi: 10.1186/s12887-021-02952-1. PMID: 34743682; PMCID: PMC8574029.
5. Diao H, Wang H, Yang L, Li T. The impacts of multiple obesity-related interventions on quality of life in children and adolescents: a randomized controlled trial. Health Qual Life Outcomes. 2020 Jul 6;18(1):213. doi: 10.1186/s12955-020-01459-0. PMID: 32631401; PMCID: PMC7336614.
6. Jarnig G, Jaunig J, van Poppel MNM. Association of COVID-19 Mitigation Measures With Changes in Cardiorespiratory Fitness and Body Mass Index Among Children Aged 7 to 10 Years in Austria. JAMA Netw Open. 2021 Aug 2;4(8):e2121675. doi: 10.1001/jamanetworkopen.2021.21675. PMID: 34436611; PMCID: PMC8391099.
7. Karmali S, Battram DS, Burke SM, Cramp A, Johnson AM, Mantler T, Morrow D, Ng V, Pearson ES, Petrella RJ, Tucker P, Irwin JD. Perspectives and Impact of a Parent-Child Intervention on Dietary Intake and Physical Activity Behaviours, Parental Motivation, and Parental Body Composition: A Randomized Controlled Trial. Int J Environ Res Public Health. 2020 Sep 18;17(18):6822. doi: 10.3390/ijerph17186822. PMID: 32962031; PMCID: PMC7560045.
8. Marsigliante S, Gómez-López M, Muscella A. Effects on Children's Physical and Mental Well-Being of a Physical-Activity-Based School Intervention Program: A Randomized Study. Int J Environ Res Public Health. 2023 Jan 20;20(3):1927. doi: 10.3390/ijerph20031927. PMID: 36767292; PMCID: PMC9915543.
9. Fulkerson JA, Horning M, Barr-Anderson DJ, Sidebottom A, Linde JA, Lindberg R, Friend S, Beaudette J, Flattum C, Freese RL. Weight outcomes of NU-HOME: a randomized controlled trial to prevent obesity among rural children. Int J Behav Nutr Phys Act. 2022 Mar 19;19(1):29. doi: 10.1186/s12966-022-01260-w. PMID: 35305674; PMCID: PMC8934465.
10. Robbins LB, Ling J, Clevenger K, Voskuil VR, Wasilevich E, Kerver JM, Kaciroti N, Pfeiffer KA. A School- and Home-Based Intervention to Improve Adolescents' Physical Activity and Healthy Eating: A Pilot Study. J Sch Nurs. 2020 Apr;36(2):121-134. doi: 10.1177/1059840518791290. Epub 2018 Aug 1. PMID: 30068245.
11. Valentini NC, Nobre GC, de Souza MS, Duncan MJ. Are BMI, Self-Perceptions, Motor Competence, Engagement, and Fitness Related to Physical Activity in Physical Education Lessons? J Phys Act Health. 2020 May 1;17(5):493-500. doi: 10.1123/jpah.2019-0532. PMID: 32303003.
12. Liang Y, Lau PWC, Jiang Y, Maddison R. Getting Active with Active Video Games: A Quasi-Experimental Study. Int J Environ Res Public Health. 2020 Oct 30;17(21):7984. doi: 10.3390/ijerph17217984. PMID: 33143064; PMCID: PMC7663622.
13. Raynor HA, Propst S, Robson S, Berlin KS, Barroso CS, Khatri P. Implementing Prevention Plus with Underserved Families in an Integrated Primary Care Setting. Child Obes. 2022 Jun;18(4):254-265. doi: 10.1089/chi.2021.0071. Epub 2021 Nov 12. PMID: 34767729; PMCID: PMC9145591.
14. Martínez-Vizcaíno V, Soriano-Cano A, Garrido-Miguel M, Cavero-Redondo I, Medio EP, Madrid VM, Martínez-Hortelano JA, Berlanga-Macías C, Sánchez-López M. The effectiveness of a high-intensity interval games intervention in schoolchildren: A cluster-randomized trial. Scand J Med Sci Sports. 2022 Apr;32(4):765-781. doi: 10.1111/sms.14113. Epub 2021 Dec 24. PMID: 34919774.
15. Zhang L, Zhu X, Haegele JA, Wang D, Wu X. Effects of a one-year physical activity intervention on fundamental movement skills of boys with severe intellectual disabilities. Res Dev Disabil. 2021 Jul;114:103980. doi: 10.1016/j.ridd.2021.103980. Epub 2021 May 10. PMID: 33984668.
16. Andrade A, Cruz WMD, Correia CK, Santos ALG, Bevilacqua GG. Effect of practice exergames on the mood states and self-esteem of elementary school boys and girls during physical education classes: A cluster-randomized controlled natural experiment. PLoS One. 2020 Jun 5;15(6):e0232392. doi: 10.1371/journal.pone.0232392. PMID: 32502164; PMCID: PMC7274370.
17. Long KZ, Beckmann J, Lang C, Seelig H, Nqweniso S, Probst-Hensch N, Müller I, Pühse U, Steinmann P, du Randt R, Walter C, Utzinger J, Gerber M. Impact of a school-based health intervention program on body composition among South African primary schoolchildren: results from the KaziAfya cluster-randomized controlled trial. BMC Med. 2022 Jan 27;20(1):27. doi: 10.1186/s12916-021-02223-x. PMID: 35081959; PMCID: PMC8793158.
18. Thomsen CF, Goharian TS, Larsen KT, Goetze JP, Andersen LB, Jeppesen JL. Intensive Lifestyle Intervention Increases Plasma Midregional Proatrial Natriuretic Peptide Concentrations in Overweight Children. J Am Heart Assoc. 2021 Jul 6;10(13):e020676. doi: 10.1161/JAHA.121.020676. Epub 2021 Jun 28. PMID: 34180245; PMCID: PMC8403313.
19. Raine LB, Kao SC, Drollette ES, Pontifex MB, Pindus D, Hunt J, Kramer AF, Hillman CH. The role of BMI on cognition following acute physical activity in preadolescent children. Trends Neurosci Educ. 2020 Dec;21:100143. doi: 10.1016/j.tine.2020.100143. Epub 2020 Oct 22. PMID: 33303110; PMCID: PMC8683838.
20. Ketelhut S, Ketelhut SR, Ketelhut K. School-Based Exercise Intervention Improves Blood Pressure and Parameters of Arterial Stiffness in Children: A Randomized Controlled Trial. Pediatr Exerc Sci. 2020 Sep 16;33(1):1-7. doi: 10.1123/pes.2020-0053. PMID: 32937598.
21. Vetter M, O'Connor HT, O'Dwyer N, Chau J, Orr R. 'Maths on the move': Effectiveness of physically-active lessons for learning maths and increasing physical activity in primary school students. J Sci Med Sport. 2020 Aug;23(8):735-739. doi: 10.1016/j.jsams.2019.12.019. Epub 2019 Dec 23. PMID: 31926869.
22. Yoshinaga M, Miyazaki A, Aoki M, Ogata H, Ito Y, Hamajima T, Tokuda M, Lin L, Horigome H, Takahashi H, Nagashima M. Promoting physical activity through walking to treat childhood obesity, mainly for mild to moderate obesity. Pediatr Int. 2020 Aug;62(8):976-984. doi: 10.1111/ped.14253. Epub 2020 Aug 10. PMID: 32304151.
23. Varagiannis P, Magriplis E, Risvas G, Vamvouka K, Nisianaki A, Papageorgiou A, Pervanidou P, Chrousos GP, Zampelas A. Effects of Three Different Family-Based Interventions in Overweight and Obese Children: The "4 Your Family" Randomized Controlled Trial. Nutrients. 2021 Jan 24;13(2):341. doi: 10.3390/nu13020341. PMID: 33498894; PMCID: PMC7911878.
24. Shang X, Li Y, Xu H, Zhang Q, Hu X, Liu A, Du S, Li T, Guo H, Li Y, Xu G, Liu W, Ma J, Ma G. Independent and Interactive Associations of Fitness and Fatness With Changes in Cardiometabolic Risk in Children: A Longitudinal Analysis. Front Endocrinol (Lausanne). 2020 Jun 12;11:342. doi: 10.3389/fendo.2020.00342. PMID: 32595599; PMCID: PMC7304437.
25. Choo J, Yang HM, Jae SY, Kim HJ, You J, Lee J. Effects of the Healthy Children, Healthy Families, Healthy Communities Program for Obesity Prevention among Vulnerable Children: A Cluster-Randomized Controlled Trial. Int J Environ Res Public Health. 2020 Apr 22;17(8):2895. doi: 10.3390/ijerph17082895. PMID: 32331366; PMCID: PMC7215792.
26. Morell-Azanza L, Ojeda-Rodríguez A, Azcona-SanJulián MC, Zalba G, Marti A. Associations of telomere length with anthropometric and glucose changes after a lifestyle intervention in abdominal obese children. Nutr Metab Cardiovasc Dis. 2020 Apr 12;30(4):694-700. doi: 10.1016/j.numecd.2019.12.002. Epub 2019 Dec 10. PMID: 32007334.
27. Logan NE, Occidental N, Watrous JNH, Lloyd KM, Raine LB, Kramer AF, Hillman CH. The complex associations between adiposity, fitness, mental wellbeing and neurocognitive function after exercise: A randomized crossover trial in preadolescent children. Prog Brain Res. 2024;283:123-165. doi: 10.1016/bs.pbr.2023.11.004. Epub 2023 Dec 19. PMID: 38538186.
28. Robinson TN, Matheson D, Wilson DM, Weintraub DL, Banda JA, McClain A, Sanders LM, Haskell WL, Haydel KF, Kapphahn KI, Pratt C, Truesdale KP, Stevens J, Desai M. A community-based, multi-level, multi-setting, multi-component intervention to reduce weight gain among low socioeconomic status Latinx children with overweight or obesity: The Stanford GOALS randomised controlled trial. Lancet Diabetes Endocrinol. 2021 Jun;9(6):336-349. doi: 10.1016/S2213-8587(21)00084-X. Epub 2021 Apr 29. PMID: 33933181; PMCID: PMC8241238.
29. Marsh S, Taylor R, Galland B, Gerritsen S, Parag V, Maddison R. Results of the 3 Pillars Study (3PS), a relationship-based programme targeting parent-child interactions, healthy lifestyle behaviours, and the home environment in parents of preschool-aged children: A pilot randomised controlled trial. PLoS One. 2020 Sep 17;15(9): e0238977. doi: 10.1371/journal.pone.0238977. PMID: 32941530; PMCID: PMC7498059.
